# Cooking for Health: a comprehensive narrative review of Culinary Medicine as an educational tool in medical training in Brazil and Globally

**DOI:** 10.20945/2359-4292-2023-0491

**Published:** 2024-07-18

**Authors:** Ana Carolina Junqueira Vasques, Caroline Dário Capitani, David M. Eisenberg, Licio Augusto Velloso, Bruno Geloneze

**Affiliations:** 1 Universidade Estadual de Campinas Faculdade de Ciências Aplicadas Limeira SP Brasil Universidade Estadual de Campinas, Faculdade de Ciências Aplicadas, Limeira, SP, Brasil; 2 Universidade Estadual de Campinas Laboratório de Investigação em Metabolismo e Diabetes Unidade de Medicina Culinária e Nutrição Campinas SP Brasil Universidade Estadual de Campinas, Laboratório de Investigação em Metabolismo e Diabetes, Unidade de Medicina Culinária e Nutrição, Campinas, SP, Brasil; 3 Universidade Estadual de Campinas Centro de Pesquisa em Obesidade e Comorbidades Campinas SP Brasil Universidade Estadual de Campinas, Centro de Pesquisa em Obesidade e Comorbidades, Campinas, SP, Brasil; 4 Harvard T.H. Chan School of Public Health Boston Massachusetts United States of America Harvard T.H. Chan School of Public Health, Boston, Massachusetts, United States of America

**Keywords:** Cooking, diet, health education, counseling, physicians, dietitians

## Abstract

The poor diet quality in line with the rising prevalence of noncommunicable chronic diseases, coupled with the substantial deficit in nutritional education within medical training programs, has precipitated the emergence of Culinary Medicine as an evolving discipline. Culinary Medicine fuses the art of home cooking with the sciences of human nutrition, psychology, gastronomy, and medicine to promote health and well-being. This comprehensive narrative review explores the diverse facets of Culinary Medicine, elucidating its historical evolution, theoretical foundations, educational initiatives in Brazil and worldwide, and its practical implementation in clinical contexts. By integrating evidence-based nutrition knowledge with culinary skills, behavior change tools, and well-established principles of healthy dietary practices, Culinary Medicine arrives to empower individuals – physicians and patients – to make informed dietary choices and enhance their overall health outcomes. Moreover, this review contemplates the roles of physicians in providing dietary guidance within the Culinary Medicine paradigm and the challenges associated with incorporating Culinary Medicine as a complementary facet of conventional medical care and medical education.

## INTRODUCTION

Malnutrition in all its manifestations – encompassing undernutrition, overweight, obesity, and other diet-related noncommunicable diseases – has assumed a pivotal role as a major contributor to morbidity and mortality across the world. According to the 2017 Global Burden of Disease report, poor-quality diet accounted for 22% of all adult deaths (1). In Brazil, according to the Surveillance System of Risk and Protective Factors for Chronic Diseases by Telephone Survey for Adults in Brazil (Vigitel), the frequency of obesity was 24.3% in 2023, considering the adult population living in Brazilian capital cities (2). While obesity continues to advance as a pressing global public health concern, malnutrition and food insecurity continue to reach unimaginable rates in the 21st century. In Brazil, a recent national survey revealed that merely four out of 10 families have unrestricted access to food, signifying that over half (58.7%) of the Brazilian populace grapples with food insecurity to varying degrees (3). Malnourished patients are also a critical point since they remain hospitalized longer and exhibit elevated morbidity and mortality rates (4).

Confronted with this epidemiological panorama, the role of nutrition in medicine has undergone a transformation, in the sense of using “food in medicine” to prevent and manage various health problems and modify the natural history of diseases (5). Over the past decade, several publications have raised concerns regarding the marginal emphasis placed on nutrition in medical curricula, to mobilize society, universities, and governmental bodies toward rectification (5-8). A US survey conducted in 2008 divulged that merely 14% of physicians felt adequately prepared to dispense nutritional guidance (9). In 2010, a study encompassing 109 medical schools in the US revealed an average of 19.6 hours of nutrition-related coursework spanning the 4-year medical program, with the majority concentrated in the early stages of the curriculum. These courses predominantly centered on fundamental aspects of nutrition, such as physiology and biochemistry, as well as the consequences of nutrient deficiencies. However, courses lacked integration with human dietary practices and chronic diseases, including guidance on how to make a healthy diet enjoyable and real-world applied, as well as strategies for successfully counseling patients on making healthy dietary changes. At that moment, the minimum requirement for nutrition instruction in medical education in the US was 25 hours, yet only 27% of the evaluated medical schools met this criterion (10,11). A recent survey by the Nutrition and Lifestyle Working Group of the American College of Cardiology’s Cardiovascular Disease Prevention Section disclosed that 73% of physicians surveyed reported receiving minimal or no education during medical residency to equip them to offer nutritional guidance to patients. In the Americas, the scarcity of nutritional education was even more pronounced, with 58% of physicians either lacking recall or encountering a notable absence of nutritional education in medical school curricula (7). In Brazil, the most recent national curricular guidelines for undergraduate medical education (12) do not include the words “nutrition”, “food”, or “diet”.

Within this overall context, Culinary Medicine has emerged worldwide as a “novel discipline” within the medical course to support nutrition knowledge and specific nutritional counseling for future physicians. Despite the enthusiasm surrounding Culinary Medicine, it remains a largely unfamiliar concept among many physicians. This comprehensive narrative review explores the various facets of Culinary Medicine, shedding light on its historical trajectory, theoretical framework, educational programs in Brazil and worldwide, and its application in clinical contexts.

## METHODS

An electronic search for articles was carried out in the databases MEDLINE/PubMed, Science Direct, ClinicalKey, and Google Scholar from August to November 2023 by two researchers (ACJV and CDC). The search terms were used either alone or linked with the Boolean operators “AND” or “OR”: (“culinary medicine”), (“teaching kitchen”), (“culinary medicine” AND “teaching kitchen” OR “home cooking” OR “culinary skills”), and (“physician” AND “self-care” OR “nutrition education”). Original studies in English and Portuguese were selected, and there was no restriction on the date of publication. The narrative review was designed according to the Scale for the Assessment of Narrative Review Articles (13).

### A brief history of Culinary Medicine

In a retrospective examination of history, Culinary Medicine electives were first offered over 130 years ago, in 1893, when the British Medical Journal printed a call for medical students to attend four sick care and convalescent cookery courses to “gain practical information on matters of such vital importance to their future patients” (14). Subsequently, as documented by La Puma (15), the inaugural cooking and nutrition elective course in the US occurred in 2003 at the SUNY-Upstate Medical University, situated in Syracuse, New York. In 2007, the inaugural edition of the conference titled “Healthy Kitchens, Healthy Lives – Caring for Our Patients and Ourselves” was directed by faculty from Harvard University. This event was the result of a collaborative effort between faculty members from the Public Health School and the esteemed Culinary Institute of America. The inclusion of culinary education in the form of cooking demonstrations and participatory hands-on cooking workshops was explored, combined with expositive nutrition-related classes (16).

In the year 2012, Tulane University created the first Culinary Medicine center in a US medical school, which was subsequently named The Goldring Center for Culinary Medicine. This pioneering initiative established a teaching kitchen within the confines of a medical institution, establishing itself as a leader in the Culinary Medicine movement (15). This milestone marked the initiation of formal culinary and nutritional education within the American medical curriculum. Presently, this movement has proliferated: over 50 universities across the US have incorporated Culinary Medicine electives into their medical curricula (12), and the movement has spread to Europe and Latin America.

In the US, reforms in both undergraduate and graduate medical education have been meticulously devised to better equip future physicians for the evolving health care landscape of the 21st century. These reforms encompass an innovative approach to medical nutrition education and training, embracing interprofessional education, an expansive array of preventive topics inclusive of Lifestyle Medicine, augmented nutritional education, and proficiency development through hands-on experimental, investigative, and clinical activities (17,18). Concurrently, in the United Kingdom, the Association for Nutrition developed the Undergraduate Curriculum in Nutrition for newly qualified doctors from the Academy of Medical Royal Colleges. This curriculum has been designed to be integrated into the core curriculum of undergraduate medical students (19).

Although Culinary Medicine still lacks a universally accepted definition, it can be aptly characterized as an emergent evidence-based medical discipline residing within the domains of Lifestyle Medicine and Preventive Medicine. Culinary Medicine embraces transdisciplinary knowledge of nutrition, psychology, and gastronomy in the science of medicine, with a focus on healthy eating, culinary skills, and a behavioral counseling approach in combination with appropriate medical care (12,16). In the contemporary era, Culinary Medicine emerges as a potential low-cost and high-impact strategy to equip medical students with practical nutrition skills that can be employed in clinical settings.

### The foundations of Culinary Medicine

**Figure 1:**
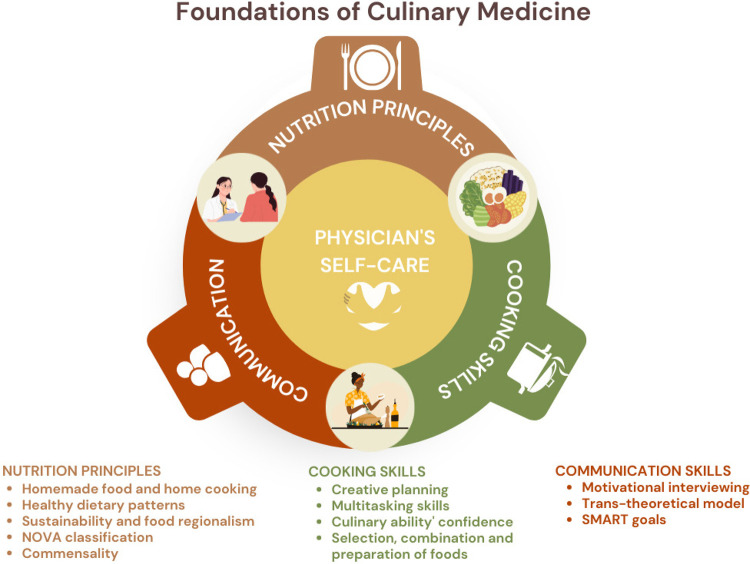
The pillars of Culinary Medicine.

Culinary Medicine can be comprehended through the lens of four fundamental pillars: physician’s self-care, culinary skills, fundamental nutrition principles, and effective communication (Figure 1). These pillars synergize within clinical practice to encourage home cooking and promote healthy eating (18). In the following sections, each of these pillars will be detailed, focusing on the purpose of each pillar and the instruments employed for their application in Culinary Medicine practice.

### Physician’s self-care

The query of whether a physician’s lifestyle correlates with that of their patients has garnered scrutiny from various research cohorts in recent decades. The collective findings consistently affirm a positive linkage between a physician’s way of life and the advice they give to their patients (20-23). Regarding dietary practices, data extracted from the Physicians’ Health Study demonstrated an association between the dietary fat intake of physicians and their tendency to recommend lifestyle modifications aimed at reducing cholesterol (24). Interestingly, patients who watched a video of a physician guiding basic dietary advice and exercise perceived the physician as more credible and inspiring if they divulged their health-related practices. This investigation concluded that physicians’ disclosure of their healthy behaviors can enhance conventional counseling methods (25).

Considering this compelling evidence, medical educational institutions should endeavor to instruct and motivate health care professionals to adopt a healthful lifestyle and communicate more effectively regarding their health habits with their patients. Within the domain of Culinary Medicine, with a focus on both training and clinical application, the emphasis is placed on bridging the gap between physicians and, subsequently, their patients, fostering healthier dietary patterns through sustainable food choices surrounding shopping, meal preparation, and eating healthy, to promote health and mitigate the incidence of food-related diseases for both (15,26).

### Culinary skills

Studies have consistently highlighted the health benefits of “home cooking” and “eating homemade food” (27-32). Within this sphere, developing or improving culinary skills involves actions in line with the facilitators and barriers to enhance “home cooking” and/or increase eating of healthy “homemade food.” Although the definition of culinary skills is not universally accepted (33-36), a systematic review conducted in Brazil recommended that culinary skills include four basic domains (36):

1. Creative planning: considers creativity in the planning and preparation of homemade meals based on *in natura* and minimally processed foods, as well as anticipation of procedures that facilitate the act of cooking.
2. Multitasking skills: comprises the ability to perform domestic tasks simultaneously with culinary practices.
3. Confidence regarding culinary skills: refers to the individual’s confidence related to the use of culinary techniques and utensils.
4. Selection, combination, and preparation of food: considers sensory aspects and food quantification to adjust purchases and culinary procedures.

A new conceptualization for culinary skills termed “culinary autonomy” was recently proposed (37). This concept denotes the ability to think, decide, and act in the preparation of home cooking, using mostly *in natura* or minimally processed foods, under the influence of interpersonal relationships, the environment, values culture, access to opportunities, and the guarantee of rights. Grounded in these frameworks – whether “culinary skills” or “culinary autonomy” – home cooking emerges as the cornerstone of a healthy diet. Nevertheless, it can run into some obstacles, related or not to the individual, which means that home cooking is a complex practice that involves more than simply preparing and consuming food.

Critical determinants and obstacles to culinary practice include gender, employment, family support network, culture, ethnicity, available time, low culinary skills, and lack of confidence (38-40). An individual’s comprehension of culinary practice is notably contingent on the contextual backdrop in which they are situated (38). Some facilitators can encourage home cooking, such as organization, planning, and enjoyment of cooking. Some basic principles are essential to address the low confidence barrier, in special culinary education intervention in the culinary skill level. Following this rationale, persons with better skills may feel more confident and consequently have more motivation to try to practice and implement home cooking (39).

**Figure 2:**
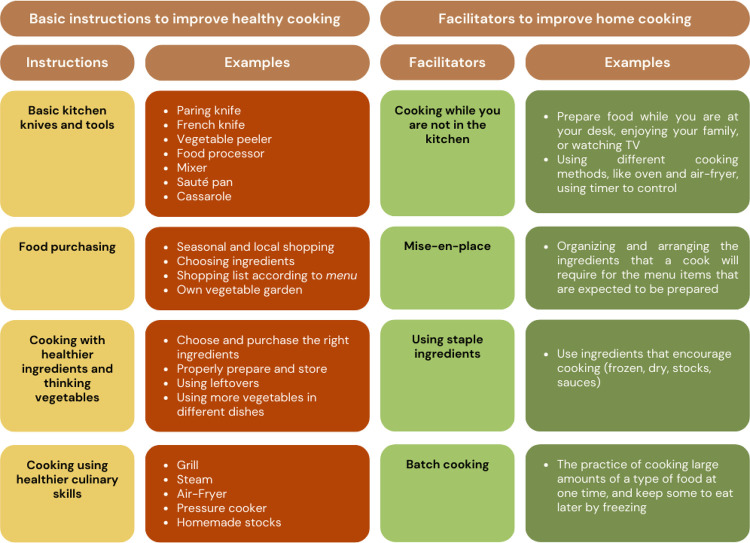
Basic instructions and facilitators for improving home cooking and healthy cooking.

There are basic instructions and facilitators to improve or develop culinary skills, which have been organized and taught to physicians to improve healthy home cooking and more eating of homemade food. Some of them are detailed in Figure 2 (36,39,41-43). Moreover, some of these culinary skills can be taught in cooking classes, including knife handling, meal-planning, grocery shopping, food budgeting, prepping and cross-utilizing ingredients, cooking techniques (searing, roasting, etc.), nutrition label interpretation, and proper food storage (41).

A final important aspect concerns the assessment of cooking skills in clinical practice. The assessment of culinary skills, ultimately, means looking at a person’s diet to ensure it is balanced and healthy and that they practice healthy cooking – which consists of preparing meals using mostly unprocessed or minimally processed foods and culinary ingredients, such as salt, sugar, oils, and fats (44,45). In the Brazilian context, some instruments for the assessment of culinary skills have been developed and validated (46-48). These instruments hold the potential to be employed across diverse settings to assess culinary skills, make diagnostics, and formulate strategies aimed at cultivating health-conscious habits among professionals and their patients concerning home-cooked meals.

The Food Guide for the Brazilian Population, a globally recognized reference document for public policies, addresses cooking skills or culinary skills as an emancipatory practice that promotes the adoption of healthy eating for individuals and communities (44). Despite seeming like a paradox of contemporary society, culinary has been identified as a strategic tool for Food and Nutrition Education (47,49-51) to promote healthy dietary habits.

### Fundamental nutrition principles

The concepts that encompass healthy eating comprehend healthy dietary patterns and commensality, intimately associated with home-cooked meals and the consumption of healthful meals. In the current setting, nutrition misinformation proliferates on social media platforms, accompanied by false promises about dietary regimens and specific nutrients (52). In this context, it is essential that physicians, as well as other health professionals, prioritize the acquisition of information from official guidelines and well-referenced scientific literature to maintain current and well-informed perspectives on nutrition.

In general, official dietary guidelines produced by each country serve as an instrument for promoting healthful eating practices at both the individual and population levels, while also contributing to policies designed to advance, support, and safeguard the health and nutritional well-being of the populace (44,53). These guidelines provide dietary patterns structured to ensure nutrient adequacy, promote cardiovascular health, and enhance overall well-being. The current guidelines consider diverse life stages, individual preferences, ethnic and religious customs, as well as cultural and social dimensions (18,54). The Dietary Guidelines for the Brazilian Population, for instance, provide a comprehensive compendium of information and recommendations on dietary practices. Some principles described in the Brazilian Guide – such as “food is more than nutrient intake”, endorsement of socially and environmentally sustainable food systems, and fostering autonomy in dietary choices (44,53) – are essential to promote healthful dietary patterns. This guideline encourages the consideration of cultural and social aspects in dietary practices, offering guidance on the act of eating and commensality while addressing contextual factors such as timing, setting, and companionship, which influence the gratification derived from food (44). Notably, the guideline pioneered the incorporation of the NOVA classification, and the “golden rule” of the Brazilian guideline is “the preference for *in natura* or minimally processed foods and freshly prepared dishes over ultraprocessed options” (44).

Evidence-based guidance for healthful dietary patterns involves food items and their constituent nutrients, emphasizing the consumption of fruits, vegetables, whole grains, healthy protein sources (including fish, seafood, legumes, and nuts), and liquid plant-based oils (*e.g.*, olive, canola, and soybean oils), food intact matrix, while urging restraint in the consumption of sugary beverages, ultraprocessed foods, processed meats, sodium-rich items, alcoholic beverages, and tropical oils in daily food preparation, such as coconut oil and palm oil, as they have a high content of saturated fatty acids with a cholesterol-increasing profile (55). Well-researched dietary patterns that confer protection against cardiometabolic disorders and promote overall health include the Mediterranean diet, the Dietary Approaches to Stop Hypertension (DASH) regimen, healthful vegetarian and plant-based diets, and the more recent Planetary Health Diet (55,56).

These dietary paradigms consistently align with healthfulness and exhibit a lower environmental footprint compared with typical Western dietary patterns (57).

### Communication

The ability to communicate nutritional information to patients, tailored to their educational and health literacy levels, is indicative of empathetic counseling and nutritional competence that reflects care. These competencies, encompassing interpersonal skills and communication, are oriented toward patient-centeredness, compassion, appropriateness, and effectiveness (18). They can be further enhanced through the utilization of specialized questionnaires designed to assess attitudes toward home cooking, self-assurance in culinary activities, confidence in employing vegetables, cooking skills, and food-related skills (58,59). Consequently, it is very important, during a medical consultation, to demonstrate the ability to access and incorporate new dietary guidelines, counseling methodologies, and diet assessment and education tools into practice. Competency in practice-based improvement includes the capacity for self-monitoring and continuous self-improvement toward achieving nutritional proficiency (18,60).

In Culinary Medicine, the most embraced behavioral tool is motivational interviewing, employing open-ended questions (61), considering the stage of behavior change followed by the establishment of culinary objectives over three months (62). These goals must adhere to the SMART goals, meaning they are Specific, Measurable, Assignable, Realistic, and Time-bound (63). SMART goals are universally recognized as efficacious management objectives, acknowledged as the gold standard for formulating effective, quantifiable goals and targets. Physicians should narrow their culinary focus down to a select few tangible objectives, ensuring they resonate with the patient’s confidence and can be accomplished within the subsequent three months (60). Ultimately, health care professionals should integrate home cooking into the intake assessment, communicate the inherent value of home-cooked meals, prescribe, and refer culinary resources, and incorporate SMART goals during encounters, with the primary objective of initially encouraging patients to prepare their meals at home (61).

### Attributions of health care professionals regarding nutritional guidance

Health dietary patterns and nutritional status are crucial aspects that may intersect with the practices of all health care professionals, particularly in the context of integrated multidisciplinary care. Indeed, nutrition is inextricable from all other therapeutic modalities, whether clinical or surgical (5,64). In a recent publication concerning the framework for food and nutrition care in primary health care, the Brazilian Ministry of Health emphasized that the responsibility for nutritional care should be a collective endeavor involving all members of the multidisciplinary health care team. Collaboration in primary health care does not necessitate the elimination of each professional category’s specific practices but rather requires different professionals to operate within the framework of a holistic approach (65).

It is widely acknowledged that each profession is subject to distinct regulations within individual countries. In Brazil, for instance, the prescription of nutrition therapy is included in the dietitians’ list of duties as professionals who are experts in the scope of Food and Nutrition care (17,66,67). Given their extensive training in nutrition and food science, registered dietitian nutritionists are ideally positioned to provide impactful instruction in Culinary Medicine and play a pivotal leadership role within this discipline (68). Physicians with enhanced nutritional training are more likely to recognize the benefits and opportunities associated with nutritional interventions, consequently facilitating more effective referrals to registered dietitians and enabling them to synchronize their communication efforts in a collective endeavor to guide patients toward dietary changes (64).

In the Health Meets Food program, some medical students initially lacked a comprehensive understanding of the role of dietitians. However, after participating in the Culinary Medicine elective, first-year students significantly increased their comprehension of the roles and contributions of dietitians to the health care team, from 37% to 93% (52). Physician appointments are great opportunities to underscore the significance of nutrition and lifestyle for optimal health, emphasizing that medications alone, while important, are insufficient. Well-trained physicians are also better equipped to advocate for public policies addressing the underlying factors contributing to poor nutrition (18,64).

Given the complexities inherent in the determinants of the health-disease continuum, collaborative practice involving professionals from diverse areas is increasingly acknowledged and imperative to render health care more effective and comprehensive (69).

### Teaching Kitchen as future classrooms for Culinary Medicine education: curricula of Brazilian and international emerging Culinary Medicine programs

Teaching kitchens, conventionally associated with nutrition and gastronomy courses, are presently gaining recognition and prominence as laboratories of the future within medical education, educational institutions, hospitals, food markets, food distribution centers, public libraries, botanical gardens, and churches, among other places (70). In 2016, the “Teaching Kitchen Collaborative”, a nonprofit organization, was established as a collaborative initiative between the Harvard T.H. Chan School of Public Health and the Culinary Institute of America. According to the Teaching Kitchen Collaborative team, teaching kitchens function as experiential learning laboratories for life skills, emphasizing a comprehensive approach to promoting lifelong healthful eating, cooking, and thoughtful dietary choices, rather than advocating for a specific “diet” (70). The Teaching Kitchen Collaborative became an independent nonprofit organization in 2020 (71). Characteristics of representative Teaching Kitchens have recently been summarized (72).

It is within these teaching kitchens that Culinary Medicine courses are predominantly delivered in numerous universities, although some institutions now favor digital platforms. These digital platforms enable professors and students to engage in culinary training within the familiarity of their domestic kitchens, promoting a closer alignment with everyday routines and resources (73,74). Recent studies suggest that teaching kitchen classes presented virtually can be as effective as teaching kitchen classes presented in person (75). Presently, the curricula of various Culinary Medicine programs and electives (Table 1) exhibit variations in content and the allocation of theoretical and practical components. Nevertheless, the core elements typically encompass fundamental aspects of nutrition, culinary skills, and behavioral interventions.

**Table 1:** Summary of curricula for primary courses and elective disciplines in Culinary Medicine in Brazil and internationally

**Location**	**Culinary Medicine Curriculum**
**Brazil**	
São Paulo University (USP) (76)	From salt intake to hypertension: physiological effects of high-salt diets Sugar and carbohydrates – metabolism and adverse effects of excessive consumption (hands-on cooking where the student can reduce the replacement of sugar and encourage the use of whole foods) Fats – physiological effects of different types of vitamins and their effects on health (hands-on cooking where the student can understand the use of fat in food preparation and possible replacements)
Federal University of São Paulo (Unifesp)*	Culinary Medicine as a health promotion tool for physicians, patients, public health, and the community Healthy eating – the basics every doctor should know Introduction to culinary techniques: Advise on skills such as food shopping, storage, and freezing; planning, organizing, and preparing meals Guide to key ingredients for the preparation of a unique dish, but with several uses Cooking on a budget Cooking with little time Types of food cuts Cooking methods Development of cooking skills through hands-on demonstration in the kitchen
School of Medical Sciences of Santa Casa de São Paulo*	Fifth-year medical students: practical workshop for foods with fat, sugar, carbohydrates, salt, and proteins Second-year medical students: the aim is to distribute and follow the curriculum of each component/disease to be worked on (such as hypertension and diabetes), removal of ultraprocessed products, removal of fat, use of unconventional food plants (UFPs), and inclusion of proteins in different ways
University of Campinas (Unicamp)*	Introduction to Culinary Medicine Diet and its impact on noncommunicable chronic diseases The relationship between food, home cooking, and homemade food with health and longevity The Dietary Guidelines for the Brazilian Population The Pillars of Culinary Medicine: Physician’s self-care Cooking skills Communication skills Nutrition principles Culinary Medicine in Pediatrics Information and misinformation in the context of nutrition and Culinary Medicine Culinary Medicine in practice
**Abroad**	
The Goldring Center for Culinary Medicine at Tulane University (77)	Condition and disease-specific modules. Fundamental dietary and nutrition knowledge with basic culinary skills through hands-on cooking classes Culinary Medicine basics and coordination of classes for the community and first-year students Hands-on culinary labs, culinary nutrition classes, and academics related to medical nutrition therapy
Stanford University (11)	Introduction to kitchen basics Sauté, stir-fry, simmer, braise Roasting Soups and salads Beans and whole grains: building healthy bowls Healthy breakfast Pastas and sauces The dessert flip Final project and potluck
Health Meets Food (78)	Introduction to Culinary Medicine Weight, obesity, and portion control Fats Food allergy and intolerance Protein and vegetarianism Kidneys and sodium Carbohydrates and diabetes mellitus The pediatric diet: a family approach to healthy children Sports nutrition Cancer nutrition: prevention and diet after diagnosis Pregnancy and nutrition Diabetes and pregnancy with reduced sugar content Celiac disease Food allergy Information on food sources, hidden allergens, and ingredient substitutions Food and neurocognition Antiinflammatory diet Inflammatory bowel disease, irritable bowel syndrome, and gastroesophageal reflux disease Congestive heart failure HIV/AIDS nutrition The geriatric diet Mindfulness and motivational interviewing Eating disorders Myths, fad diets, supplements, and controversies Polycystic ovary syndrome Bariatric diet Food safety and sanitation Billing and coding for lifestyle medicine Phenylketonuria nutrition Enteral and parenteral nutrition
European project Dr.PED-Chef (73)	Fundamentals of healthy nutrition for primary health care practitioners Useful resources and effective tools for promoting healthy eating for children, adolescents, and their families Introduction to Culinary Medicine Health communication and counseling on promoting healthy eating Health communication and counseling on weight management of children/adolescents at increased risk of obesity Culinary Medicine in practice

*The information provided was acquired through direct communication with the individuals responsible for the Culinary Medicine courses at their respective universities.

**Figure 3:**
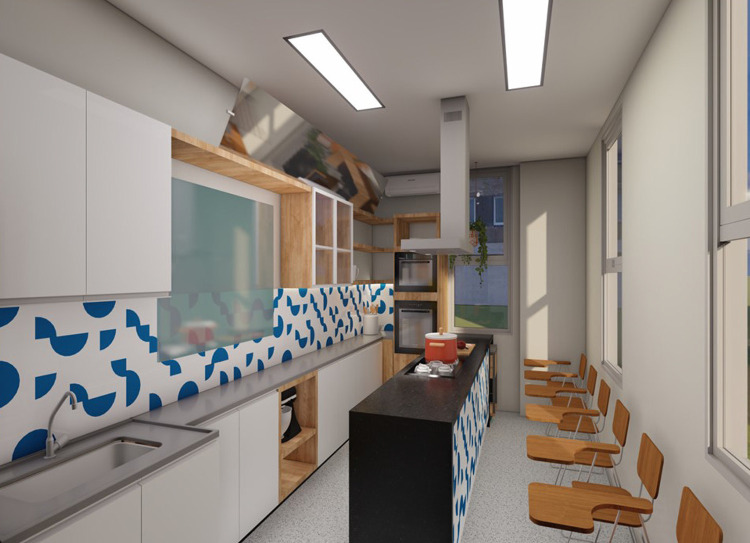
Culinary Medicine and Nutrition Unit, State University of Campinas, Brazil.

In Brazil, the Culinary Medicine elective was created at the University of São Paulo in 2018, offering hands-on cooking experiences to students across different campi (76). The Federal University of São Paulo introduced its Culinary Medicine elective in 2021, incorporating a theoretical foundation alongside practical culinary training. In 2022, the School of Medical Sciences of *Santa Casa de São Paulo* introduced a practical workshop for fifth-year medical students, and beginning in 2024, they plan to initiate a Culinary Medicine elective for second-year physician students, encompassing both theoretical and practical components. At the State University of Campinas (UNICAMP), our research group pioneered the introduction of Culinary Medicine classes in 2022, targeting fifth-year medical students. The classes take place at the Culinary Medicine and Nutrition Unit within the Laboratory of Investigation on Metabolism and Diabetes, situated at the Faculty of Medical Sciences at UNICAMP (Figure 3). This initiative emerged as an integral component of the Culinary Medicine and Nutrition (MeNu) project, which seeks to integrate nutritional principles in a transdisciplinary manner, emphasizing behavioral modification and the preparation of homemade meals. Initially, the Culinary Medicine classes were conducted in 4-hour theoretical-practical sessions, serving as a pilot project for the development of an ample 30-hour elective course delivered over 15 weeks. This course is facilitated by a multidisciplinary team of instructors, including endocrinologists, psychiatrists, dietitians with expertise in clinical nutrition, dietary techniques, and pediatrics, a gastronomy specialist, and a media and social communication professional.

Internationally, the Goldring Center for Culinary Medicine at Tulane University was the pioneer in offering Culinary Medicine training to medical students. Presently, the institution offers a comprehensive curriculum in Culinary Medicine, characterized by a rigorous workload. In addition to training for future physicians, the Goldring Center for Culinary Medicine teaches continuing medical education classes for existing medical professionals as well as free nutrition-focused cooking classes for community members. The specific content of the curriculum varies depending on the student’s stage in the medical program (77).

At Stanford University, Culinary Medicine classes are conducted within the pedagogical kitchen of the medical school. Notably, Stanford has played a pioneering role in sharing its Culinary Medicine training curriculum with the broader academic community, providing it free of charge (11).

Another initiative, the Health Meets Food, offers continuing medical education courses in Culinary Medicine to health care professionals in Washington, DC, and collaborates with partners across the US (78). This training is also available in a hybrid format to students at George Washington University, combining hands-on instruction in a professional kitchen with independent cooking experiences in their homes. Students acquire a foundational understanding of Culinary Medicine and the fundamentals of healthy nutrition for both disease prevention and treatment, all while considering issues of food equity and insecurity (79). The comprehensive training program at Health Meets Food comprises 60 hours of instruction and typically spans approximately 2 years (78).

In Europe, an interesting initiative called Dr.PED-Chef provides specialized training in Culinary Medicine to pediatric specialists. The curriculum comprehends various modules addressing nutrition-related topics, employing a combination of hands-on nutrition science and Culinary Medicine education. Notably, this program is accessible online, free of charge, and can be pursued in six languages. The project, which boasts participation from the University of Navarra in Spain and several other institutions across five countries, underscores the global relevance of Culinary Medicine (73).

In this diverse landscape, Culinary Medicine courses are characterized by hands-on learning and exhibit considerable variation in structure, learner level, and duration. Many of these programs originate from university centers, standardizing core curriculum components. Table 1 shows a detailed overview of these courses and elective disciplines in Culinary Medicine, both in Brazil and internationally.

### Impacts of Culinary Medicine on physicians’ lifestyles and the health care of their patients

In the context of the transdisciplinary nature of Culinary Medicine, a pertinent inquiry emerges: To what extent does longitudinal integration and interprofessional education within the curriculum influence the health outcomes of health care professionals and/or their patients (*e.g.*, dietary habits, physical activity, and the risk of chronic diseases)?” (43) Such discussion has also been taking place with other health professionals, like dietitians (80) and professionals who work with food, like gastronomy specialists, and will be detailed below (81).

In 1996, the CADRE Study Group (82) investigated whether educational and prompting interventions for physicians could enhance their dietary counseling of patients and subsequently induce favorable alterations in patients’ dietary habits and cholesterol levels. This multicenter, randomized, placebo-controlled trial involved 130 internal medicine residents and 254 adult outpatients with blood cholesterol levels ranging from 240 to 300 mg/dL. Resident physicians’ knowledge, attitudes, and self-reported behaviors were assessed before the intervention and 10 months later using chart audits and questionnaires. Residents’ behaviors were also assessed through exit interviews with patients. Although significant changes in patients’ cholesterol levels were not observed, the educational program notably increased the confidence levels of physicians in delivering effective dietary counseling, doubled the frequency of dietary counseling by physicians, and heightened the likelihood of patients making earnest efforts to amend their dietary practices. The authors discussed that lifelong eating habits are hard to change, perhaps even harder for older patients like the ones in their study. However, the intervention was successful in changing several intermediate outcomes, advancing patients along the stages of change.

The impact of Culinary Medicine on physicians’ health and their guidance to patients were evaluated by Eisenberg and cols. (16). An anonymous survey was completed at baseline and 12 weeks later at the conference “Healthy Kitchens, Healthy Lives – Caring for Our Patients and Ourselves.” The authors described changes in personal and professional nutrition-related behaviors reported by participants. A total of 265 (66%) respondents were physicians. The respondents reported positive changes in the frequency of cooking their own meals, improvements in their ability to provide effective nutritional guidance to patients with overweight or obesity, and enhancements in their consumption of nuts, fruits, and overall diet quality. The inclusion of “culinary education” in the form of cooking demonstrations and hands-on cooking, seems to be a potential tool to promote positive changes in both personal and professional nutrition-related behaviors. The impact on the patient’s behaviors and clinical outcomes was not evaluated by the authors. Despite the changes, the study presented some limitations, including the modest sample size and the anonymous nature of the survey.

Monlezun and cols. (2015) conducted a cross-sectional study with 627 medical students, using a 59-question survey to evaluate students’ diet, attitudes, and competencies. They investigated the superiority of hands-on cooking practices and nutrition education for preventive medicine. The results were evaluated using conditional multivariate logistic regression. The elective-implemented curriculum produced superior improvements in medical students’ training to provide patients with nutrition counseling when compared with traditional clinical education. The hands-on cooking practices also improved the diet of medical students (83).

Using the facilities from The Goldring Center for Culinary Medicine (GCCM), a pilot, randomized, controlled trial allocated 27 patients with type 2 diabetes between a control and a GCCM arm (84). Hands-on cooking and nutrition classes were taught by registered dietitians, chefs, physicians, and medical students. The six-module cooking and nutrition curriculum translates the Mediterranean diet for culture-specific kitchens across different socioeconomic levels. The control group received the standard of nutrition education, consisting of a one-time registered dietitian counseling visit with a referral opportunity to a diabetes education class certified by the American Diabetes Association. Compared with the control group, the GCCM group had greater reductions in diastolic blood pressure (p = 0.037) and total cholesterol (p = 0.044) and a superior but nonsignificant reduction in glycated hemoglobin (HbA1c) level (0.4% *versus* 0.3%, p = 0.575). There was also a greater proportion increase (though not significant) of GCCM subjects compared with controls who mostly believed they could eat correct portions (84).

In the same perspective, Monlezun and cols. (85) evaluated that hands-on cooking and nutrition education significantly improved all aspects of nutritional counseling competencies and attitudes toward a positive impact of nutritional counseling in clinical practices by medical students (n = 3,248 medical trainees). This was a 5-year, prospective, multisite, cohort study including any medical student responder, controlled by school curriculum (n = 20 medical education institutions). The students’ competencies were evaluated using 25 competency topics. The hands-on cooking practices and nutrition education improved students’ readiness to improve health outcomes, especially for cardiovascular disease.

Recently, Asher and cols. (43) published a scoping review of 33 studies, intending to map Culinary Medicine programs provided to different professionals who have the potential to influence health behavior change. The authors observed heterogeneity in the interventions. In summary, the findings of the review indicated great improvement in culinary knowledge, self-efficacy for healthier cooking, and healthier dietary patterns after Culinary Medicine training programs for medical students.

Considering that Culinary Medicine is a new emerging area of medicine, studies on its effectiveness are recent. Additionally, the vast majority of the studies are limited to the US and demonstrate favorable impacts in the short term. Upcoming studies may aim to use validated assessment instruments and examine the long-term impacts of Culinary Medicine on the (A) personal dietary and self-care behaviors of health trainees and health professionals, (B) behaviors of patients cared for by health professionals with training (and competencies) in Culinary Medicine, and (C) behaviors, health outcomes, and costs of health care of those patients (or employees, students, retirees, etc.) participating in future teaching kitchen and/or Culinary Medicine programs. These studies will need to be replicated, scaled, and validated across an array of settings, populations, countries, and cultures.

## Final message

In the worldwide epidemiological context of poor diet quality and high prevalence rates of chronic diseases, Culinary Medicine has emerged as a body of knowledge to fill the gap in medical nutrition training, improving physicians’ communication skills for patient counseling for the adoption of more home cooking and a healthy diet pattern. In line with the foundations of the four pillars of Culinary Medicine – physician’s self-care, culinary skills, fundamental nutrition principles, and effective communication – it is imperative that lifestyle transformations get started with the physician to influence patients efficaciously.

In this regard, Culinary Medicine has been acknowledged as a prescription for improved health, gaining traction within medical curricula both in Brazil and across the globe. It has yielded positive outcomes grounded in scientific evidence, with registered dietitians occupying central roles in its implementation. It can be suggested that Culinary Medicine represents the future of fundamental nutritional education for all health care professionals and offers a unique opportunity for collaboration among physicians, nutritionists, psychologists, nurses, and culinary experts, all dedicated to advancing patient health.
